# How a Training Program Is Transforming the Role of Traditional Birth Attendants from Cultural Practitioners to Unique Health-care Providers: A Community Case Study in Rural Guatemala

**DOI:** 10.3389/fpubh.2017.00111

**Published:** 2017-05-19

**Authors:** Sasha Hernandez, Jessica Bastos Oliveira, Taraneh Shirazian

**Affiliations:** ^1^Saving Mothers, New York, NY, USA

**Keywords:** traditional birth attendant, Guatemala, training model, rural HEALTH, Indigenous health, maternal mortality, low resource settings, low- and middle-income countries

## Abstract

In low- and middle-income countries (LMICs), where the rates of maternal mortality continue to be inappropriately high, there has been recognition of the importance of training traditional birth attendants (TBAs) to help improve outcomes during pregnancy and childbirth. In Guatemala, there is no national comprehensive training program in place despite the fact that the majority of women rely on TBAs during pregnancy and childbirth. This community case study presents a unique education program led by TBAs for TBAs in rural Guatemala. Discussion of this training program focuses on programming implementation, curriculum development, sustainable methodology, and how an educational partnership with the current national health-care system can increase access to health care for women in LMICs. Recent modifications to this training model are also discussed including how a change in the clinical curriculum is further integrating TBAs into the national health infrastructure. The training program has demonstrated that Guatemalan TBAs are able to improve their basic obstetrical knowledge, are capable of identifying and referring early complications of pregnancy and labor, and can deliver basic prenatal care that would otherwise not be provided. This training model is helping transform the role of the TBA from a sole cultural practitioner to a validated health-care provider within the health-care infrastructure of Guatemala and has the potential to do the same in other LMICs.

## Introduction

The majority of mothers from low- and middle-income countries (LMICs), specifically in rural settings, continue to seek and prefer traditional birth attendants (TBAs) over clinics and hospitals for care during pregnancy and delivery (1). Thus, TBAs are important to both the local community and national health infrastructure as their training and integration in the current health-care system can help improve maternal and neonatal outcomes in LMICs (2–4). Despite the significant role TBAs hold there are limitations, as few sustainable training programs exist that properly train TBAs in how to provide basic prenatal care, detect early complications, or refer high-risk pregnancies appropriately. The lack of sustainable interventions leads to major discrepancies between the skills of individual TBAs and adversely affects the care of expectant women in LMICs. The purpose of this community case study is to describe a training program for TBAs in rural Guatemala that is redefining their role as health-care providers, integrating them into the current health-care system, and increasing access to basic prenatal care for the women they serve *via* a collaborative and sustainable approach.

## Background and Rationale

Significant worldwide progress has been made toward lowering rates of maternal mortality in the last two decades (5). Yet maternal mortality ratios (MMRs) in the developing world, especially in rural regions, continue to be unacceptably high (6). This holds true in Guatemala where the national average MMR of 88 maternal deaths per 100,000 births (5) does not reflect the major disparities that exist between rural and city births and, more importantly, between indigenous and non-indigenous women (ladinas). The MMR for Mayan women is more than twice that of ladinas (163 per 100,000 compared to 78 per 100,000) (7).

One of the largest contributing factors to the difference in MMRs is that up to 80% of Mayan women deliver at home without ever receiving prenatal care due to limited access to essential obstetric care (8). In the rare event that a Mayan woman has access to higher levels of care a home birth is still preferred as health facilities present many challenges. There is a clear language barrier at predominantly Spanish-speaking hospitals as many Mayans do not speak fluent Spanish (9). Additionally, national hospitals often prohibit Mayan practices such as traditional steam baths, massages, and plant-based remedies (10). The strong adherence to cultural practices, lack of access, and distrust of health facilities causes the majority of Mayan women to rely on a Guatemalan TBA (comadrona) during labor and pregnancy (10).

Guatemala comadrona culture is unique as it is founded on a belief that their abilities are a direct gift from God revealed to them in a dream or vision that must be accepted as fate (11). Once their calling is accepted they become respected elder women in the community and are constantly sought out during pregnancy and childbirth to counsel on common problems during pregnancy, provide spiritual guidance, abdominal massages, and traditional hot steam baths (11).

Comadronas hold a unique relationship with Mayan women, and their comprehensive training can improve care during pregnancy and childbirth yet this potential role has historically been rejected by the national health system (12). Not until recently has there been some national efforts to help integrate comadronas into the health-care system by providing licenses to comadronas that attend national monthly trainings (13). During these meetings, national health staff teach about signs and symptoms of danger that require referral during pregnancy (13). Problems exist with this national monthly training program as classes are often canceled for months at a time due to shortage of training staff, and there are no data collected that measures how well comadronas are retaining and applying knowledge learned at meetings.

Ultimately, a sustainable, culturally appropriate, and comprehensive training program for TBAs that can be incorporated by national health programing should be used to help meet the health-care needs of women in rural Guatemala and other similar LMICs.

## Description of the Case

The case study presented here is the School of POWHER training program for TBAs. The School of POWHER, which stands for Providing Outreach in Women’s Health and Educational Resources, was founded after recognizing the importance of training comadronas to become experts in basic prenatal care and safe labor. The goal of the School of POWHER is to train comadronas in focused maternal health-care while respecting local customs with appropriate obstetric referral as the key to decreasing maternal death and complications in the region. To improve acceptability of the intervention, and to improve sustainability, comadronas were trained to ultimately be the trainers of the School of POWHER.

The development of the School of POWHER began in 2011 after 3 months of initial community surveying by a physician assistant educator and recognition of the unique health-care needs of Mayan women in rural Guatemala. After the importance of comadrona training was identified, WHO guidelines were considered (14) and a thorough literature review of other TBA training programs as well as a review of other training curriculum used in Latin American countries was completed. Ultimately, the curriculum was the result of a dynamic process shaped by a relationship with International Medical Relief Fund midwives that implemented a successful program for TBAs in rural El Salvador (15), the health practices of comadrona elders in Lago Atitlan Guatemala, the Saving Mothers medical team headed by the physician assistant educator, and a local Mayan physician who translated the cultural practices of her community. All lecture and clinical topics reflect current WHO and Guatemalan health-care guidelines and were approved by the local branch of the Guatemalan Ministry of Public Health and Social Assistance.

## Setting

The School of POWHER is based out of the rural department of Sololá, which is nestled in the Western Highlands of Guatemala. This region is 96.5% indigenous, has 10,051 births per year, and only 2.7% of all consults/visits in the region’s health facilities are for prenatal care (16). These demographics are reflective of rural Guatemala as a whole as the country’s poverty and low health-care access is concentrated in the rural communities mainly occupied by indigenous Mayans (17). In early 2017, an innovative primary care health model, the Inclusive Health Model (*Modelo Incluyente de Salud* or MIS in Spanish), was launched in select departments in Guatemala (18). This primary health-care model aims to provide the necessary health care for the rural populations, but more importantly, incorporates TBAs and traditional healers alongside doctors and nurses intending to bridge the gap between indigenous people and the health-care system (19). This recent shift in health-care delivery recognizes the importance of integrating indigenous health facilitators within the health-care infrastructure in a standardized way (20), a model in line with our School of POWHER training program presented in this case study.

## Collaborative Partnerships

Saving Mothers is a United States-based NGO dedicated to eradicating preventable maternal deaths and birth-related complications in LMICs. Their efforts are predominantly focused on providing education and training to local health-care providers on maternal and reproductive health. Saving Mothers has been working in the Western Highlands of Guatemala since 2009, focusing in the rural communities in the department of Sololá due to the high MMR in this region (16, 21). In 2014, Saving Mothers established its main educational intervention in Guatemala, the School of POWHER training program for comadronas. The aim for this educational program is to provide a sustainable solution for the lack of access to prenatal care and safe labor while respecting local customs and traditions. Funding for the School of POWHER, made available through small international grants and private donors, will continue to support the training program for a minimum of five more years and/or until the model is incorporated by national health programming.

The Guatemalan Ministry of Public Health and Social Welfare (MSPAS) aims to provide social assistance to all its people and therefore must carry out prevention, promotion, recovery, rehabilitation, and any necessary complementary activities to provide its people with the most complete physical, mental, and social well-being (22). The regional branch of the MSPAS in Sololá partners with Saving Mothers to help integrate education initiatives for health providers into the current health-care infrastructure. Specifically, they assist in the recruitment of comadronas for the School of POWHER, pair each comadrona student with an MSPAS auxiliary nurse at ministry health-care facilities throughout the region, and aid in the execution of any ongoing Saving Mothers research or clinical initiatives. The objective of this partnership, in line with the current primary health-care model, is to respect, support, and integrate cultural practitioners into current infrastructure to better the health of Mayan women and children.

## Methodological Aspects

From 2014 to 2017, the School of POWHER training program has had three graduating classes with a total of 48 trained comadronas. Three months prior to the start of the School of POWHER, every student was screened and selected after thorough community surveying with elder comadronas by a physician assistant-educator, also the Saving Mothers Guatemala Program Director, and input from the local branch of the MSPAS. This twofold approach ensures that the community is identifying elder comadronas that are respected and also known to MSPAS staff due to their involvement in monthly licensure meetings. Ideally, each comadrona selected speaks moderate to fluent Spanish, has her MSPAS license, is relatively young (preferably 55 years old or younger), can commit to the time intensive school schedule (only two absences are allowed in 3½ months of training), and has at least four active patients.

Once the students are identified and enrolled, usually 15–20 students per class, the School of POWHER runs for 14 weeks and teaches comadronas how to perform focused prenatal care, how to refer appropriately, tests their knowledge retention via three exams, and assesses their clinical skill improvement in real time. This immersive intervention is based on a two-pronged method composed of a 28-module didactic and a separate clinical constituent designed to incorporate all literacy levels and delivered by a certified physician assistant-educator with 10 years of experience working in the field of OB/Gyn at a large teaching hospital in the United States and in rural settings in LMICs. The School of POWHER also has a collaborative approach as a portion of the lecture arm of the curriculum is delivered by elder comadronas well versed in ancestral practices such as abdominal massages and natural remedies.

Two afternoons a week are dedicated to the 3-h intercultural didactics which have an emphasis on recognizing signs of referral for mom and baby, prenatal care, and initial management of postpartum complications (Table 1). These 28 lectures are presented in a multifaceted way including lectures, group work, role-playing, case studies, and tutorials. The clinical arm of the school ensures that the comadronas refine their traditional midwifery abilities while learning new skills under the supervision of a clinical preceptor. During monthly home visits, the comadronas practice counseling the mother about tetanus vaccines, measuring blood pressure, using a fetal Doppler, and correctly estimating delivery date. A minimum of 25 recorded prenatal visits, 1–3 supervised births, and 3 postpartum visits are required of each student during the school.

**Table 1:** **The School of POWHER curriculum overview**.

	Topic
**Week 1**	
Day 1	An Overview of Maternal-Infant Care
Day 2	The Role of the Birth Attendant, Health Care Resources, and the Emergency Plan

**Week 2**	
Day 3	Sexual Organs, Sexual Desire, and Menstruation, Ovulation, and Fertilization
Day 4	The Natural Development of Pregnancy

**Week 3**	
Day 5	The Choice of Motherhood, The Importance of Prenatal Care—Part 1
Day 6	The Importance of Prenatal Care—Part 2

**Week 4**	
Day 7	Pregnancy Complications, Part 1
Day 8	Pregnancy Complications, Part 2

**Week 5**	
Day 9	Signs and Symptoms of Danger during Pregnancy
Day 10	Case Studies on Prenatal Care, Exam 1

**Week 6**	
Day 11	Review Exam 1
Day 12	Tetanus Vaccine, Introduction to Stages of Labor

**Week 7**	
Day 13	Uncomplicated Labor: Stage One and Two
Day 14	Uncomplicated Labor: Stage Two and Three

**Week 8**	
Day 15	Review of Stages of Labor, Basic Care of the Newborn
Day 16	Complications during Labor and Delivery

**Week 9**	
Day 17	When to Refer during Labor
Day 18	Neonatal Resuscitation, Immediate Post-Partum Care, Maternal Lactation

**Week 10**	
Day 19	Maternal Lactation Review, Post-Partum Care: Day 1 & 4
Day 20	Review of Normal and Complicated Labor, Exam 2

**Week 11**	
Day 21	Medicinal Plants, Sharing of Experiences during Labor
Day 22	Infection Prevention and Management, Sterilization of Birth Tools

**Week 12**	
Day 23	Family Planning Methods
Day 24	Case Studies: Family Planning, Self Breast Exam

**Week 13**	
Day 25	Vaginal Infections and STIs
Day 26	Nutrition and Malnutrition

**Week 14**	
Day 27	Complicated Labor
Day 28	Cumulative Review, Final Exam

Each woman that completes the School of POWHER training program receives a stethoscope, blood pressure equipment, a fetal Doppler, prenatal vitamins, and safe birthing kits funded by international grants and private donations. More importantly, each comadrona is integrated into the current network of School of POWHER graduates and continues to receive monthly education, prenatal vitamins for all their patients, and safe birth kits for each birth they attend.

## Theoretical Framework

The School of POWHER was developed around the “rational model” also known as the “knowledge, attitudes, practice model” as outlined in “Health education: theoretical concepts, effective strategies, and core competencies” (23). This theoretical framework is based on the premise that increasing a person’s knowledge will induce behavioral changes (23) and was deemed the most culturally appropriate as the comadronas hold such a high-stake position in their communities and have so much influence over the health of their patients. At the core of the School of POWHER is community health education and if the comadronas begin to change their attitudes and beliefs, for example, of when it is necessary to refer during pregnancy, this change in attitudes and behavior will improve the health of their patients. By influencing both individuals’ capacities and providing environmental support, in this case through the integration into the current health system, a meaningful and sustained change in the health of individuals and communities can occur (23).

## Teaching Framework

The teaching framework for the School of POWHER follows the guidelines set forth by the Health Professions Networks Nursing & Midwifery Human Resources for Health in “Framework for Action on Interprofessional Education & Collaborative Practice” (24). This teaching framework outlines that education collaborations should occur between two or more professions in order to improve health outcomes (24). These partnerships should (1) include in-depth intercultural exchanges as well as (2) collaborative clinical practice (24). The curriculum presented in this community case study reflects these teaching guidelines. The didactic portion involves exchanges between the physician assistant educator, elder comadronas well versed in ancestral practices, and the comadrona students themselves. The clinical curriculum component reflects the importance of collaborative clinical practices as each participant is paired with an MSPAS auxiliary nurse at the national health clinics during part of her clinical training. These collaborate teaching strategies, when integrated into the current health-care system, strengthen health systems, and ultimately improve health outcomes (24).

## Methodological Adaptations for Sustainability

The ultimate goal of School of POWHER is to have a sustainable training program for comadronas taught by comadronas. This train-the-trainer model is key as improvement in a health-care workforce is largely based on training programs that can identify proficient health-care providers and prepare them as trainers (25). In working toward this, eight School of POWHER graduates from the first class continued their training in basic maternal health with Saving Mothers. They have learned the School of POWHER curriculum in depth (Table 1) through their work as teaching assistants during the second and third classes of the School of POWHER. Two head comadronas, graduates from the original class, have been identified as lead educators that will deliver the lecture portion of the school in Mayan dialect. Six additional graduates have been selected as clinical preceptors that will teach prenatal home visits during the clinical portion of the curriculum. During the 2017 School of POWHER class, there will also be a senior medical student on ground to aid in the transition, modification, and execution of the School of POWHER. The senior medical student is receiving training at a large teaching institution in the United States and has 5 years of experience in implementing educational programming in LMICs.

**Figure 1:**
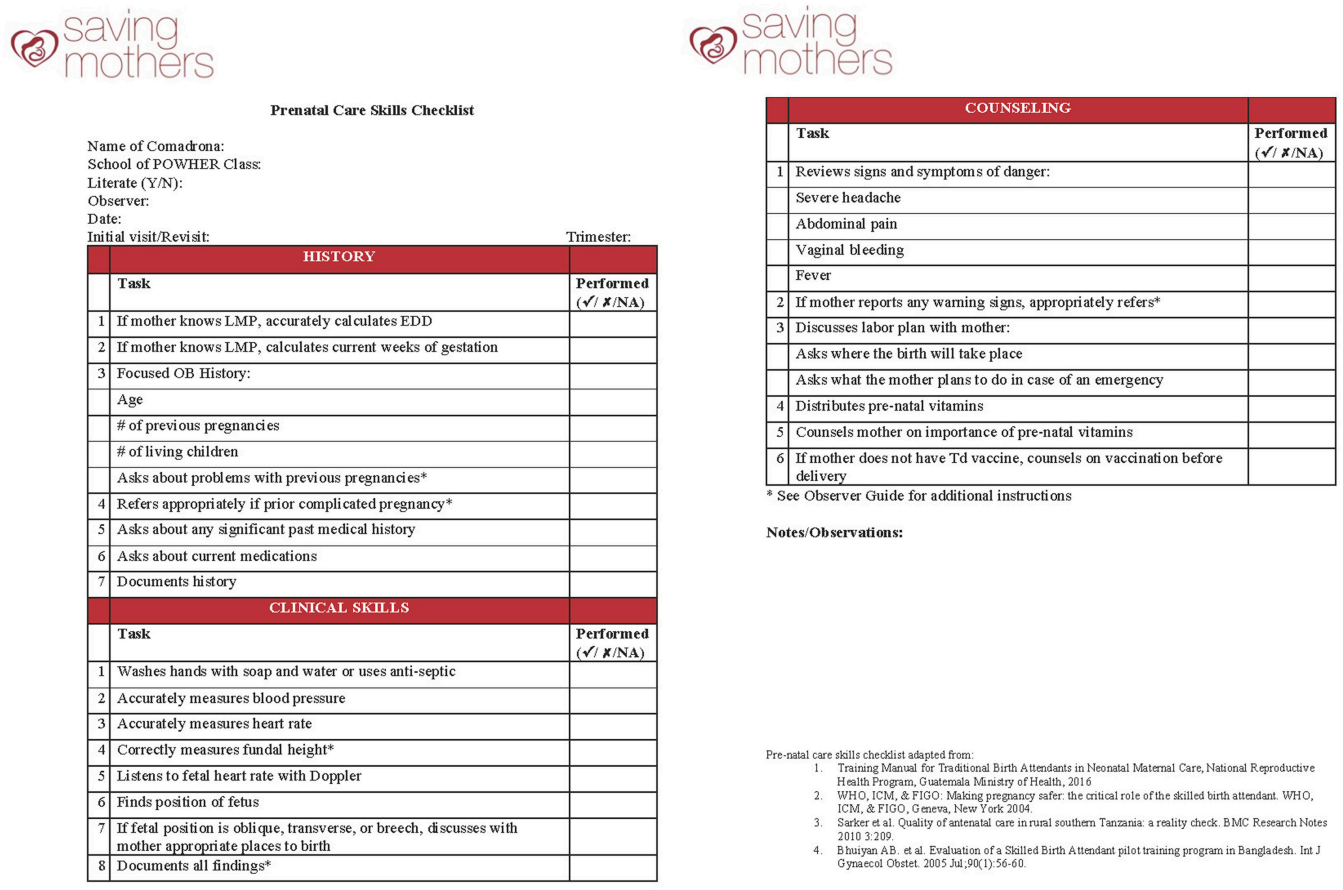
**Clinical assessment tool**.

In addition to modifying who delivers the educational intervention, future classes of the School of POWHER will incorporate a new a real-time clinical skills assessment. These clinical skills will be assessed with a prenatal skills checklist (Figure 1) that was developed from WHO health-care practices (14), other successful TBA training programs (26, 27), and whose contents have been approved by the MSPAS. In order to ensure that the prenatal clinical skills checklist was culturally relevant and an appropriate clinical tool within the School of POWHER curriculum, a pilot study testing its contents was completed from January through March of 2017. During this pilot phase, 18 graduates were assessed, 84 home visits were observed, and its final contents were adapted based on our study pilot results to modify aspects of the emergency birth plan.

With the conclusion of the pilot study demonstrating that the clinical skills checklist is culturally appropriate and relevant within the School of POWHER, the prenatal skills checklist will be administered prior to starting the curriculum and after completion of the program. This data will directly measure the clinical improvement of each comadrona as a direct result of their participation in School of POWHER and gage the level of clinical competency of participants. The clinical skills checklist not only provides a way to directly measure clinical skill development before and after the School of POWHER but also provides an accessible tool for the clinical preceptors to use as they evaluate ongoing clinical skills development of participants.

## Evaluations

All School of POWHER participants have been assessed in their clinical knowledge and retention on prenatal care, basic obstetric practice, and emergency management. This is quantitatively measured *via* multiple choice exams. During the School of POWHER, the 28 lectures are divided into 3 broader modules, and on the first day of each module, every student received a pre-test that was repeated the last day of that module. Knowledge retention is also measured 1 year post graduation. Qualitative evaluations were performed *via* surveys and informal discussion sessions at the end of the School of POWHER. At monthly follow-up meetings, patient prenatal and birth data and number and reason for referrals are collected from each participant and reported to the local MSPAS staff. In addition to all aforementioned evaluations, all future classes of the School of POWHER will have their clinical skill knowledge and retention measured before and after the educational program.

## Results

Participants in the School of POWHER educational intervention have demonstrated an improvement in multiple basic obstetrical knowledge areas such as anatomy, physiology, and pregnancy diagnosis/management (Table 2). They have strengthened their knowledge in areas of Newborn Assessment/Care, Labor & Delivery, and Family Planning (28). At 1-year follow-up of the first graduating class, basic obstetrical knowledge remained improved from the original pre-test knowledge scores (Table 2).

**Table 2:** **Mean test scores for School of POWHER class of 2014 students**.

Topic	Pre-test (%)	Post-test (%)	1-year test (%)
**Knowledge test**			
Anatomy/physiology	53	70	64
Pregnancy diagnosis	35	83	70
Prenatal care	35	70	91
Emergencies	66	86	94
Labor and delivery	68	88	78
Postpartum care	52	81	55
Newborn care	74	79	86
Sterile techniques	62	86	70
Infection prevention	62	86	70
Baby care	90	100	100
Prevention	62	76	91

Total knowledge score	59	82	79

When participants were surveyed upon completion of the educational program, they reported a new interest in continuing their education and increased confidence in themselves due to their newly learned skills. During the informal discussion sessions, the participants largely discussed the need for the training they were receiving and positively reacted to the structure/curriculum of the School of POWHER. Some of the comments reflecting the participants’ assessment of the educational intervention included:

Nobody ever thinks about comadronas. I’ve never been a part of a program like this and I’m touched that we are being involved.

The way we are learning, through group work and cases, is new for me but I have found it very helpful.

I am excited to take blood pressure and learn how to listen to the baby’s heart.

We are creating a sisterhood of comadronas, students and teachers coming together to help our community.

The training of comadronas has also significantly increased the number of women who receive prenatal care and timely referrals. In 2016, the School of POWHER participants performed 360 recorded prenatal visits and identified 15 women at risk of complicated labor, which were referred to appropriate higher level of care.

The clinical skills checklist pilot study highlighted areas of clinical strengths in the School of POWHER participants. From the 84 prenatal home visits, 56% of the visits were considered complete (at least 75% of the checklist was accurately completed). A total of 30 women were referred (6 for a previous complicated pregnancy, 4 for fetal malposition, and 20 for reporting one of the signs or symptoms of warning including fever, abdominal pain, hemorrhage, or severe headache); one of these referrals revealed fetal demise. Overall skill strengths were found in correctly taking basic vitals (correct blood pressure measurement in 76/84 visits) and providing adequate counseling (Table 3).

**Table 3:** **Type of counseling discussed during prenatal home visit in skills pilot study**.

Type of counseling	# of visits counseling occurred/total visits	# of visits counseling did not occur/total visits
Discussion of fever	40/84	44/84
Discussion of severe headache	56/84	28/84
Discussion of epigastric pain	56/84	28/84
Discussion of hemorrhage	53/84	53/84
Discussion of important of Td vaccine	64/84	20/84
Discussion of importance of prenatal vitamins	60/84	25/84

Skill weaknesses were evident in lack of hand washing (during 55/84 visits, no hand washing occurred) and lack of a discussion of an emergency birth plan (36/84 women were not asked about their plan of emergency during labor). These areas highlighted the need for further curricular emphasis on topics of hand washing and emergency birth plan during future classes of the School of POWHER. Additionally, three comadronas were identified to be consistently taking blood pressure incorrectly and were appropriately retrained.

## Discussion

This community case study presents a sustainable method of training TBAs in order to increase access to basic health care for indigenous women and improve their pregnancy and labor outcomes in a rural community in Guatemala. While this case study is novel in its educational delivery approach of using trained TBAs to teach other TBA participants, other successful programs have informed this intervention.

When training is successfully implemented in rural communities, TBAs increase their basic obstetric knowledge, are equipped for safe home deliveries, and are able to identify problems requiring referral; factors that markedly improve obstetrical outcomes (29). TBAs that go through these training programs report increased job satisfaction, are more motived to continue improving their skills (30), and have improved knowledge, attitudes, and behaviors (31). Recent systematic reviews have identified that successful programs are those that can be integrated into an existing health-care system, continue skill development (monthly or bi-monthly) of its participants for at least a year, and provide access to birth kits and resuscitation equipment (3, 4, 31).

These factors of success reported by other programs are also what makes the School of POWHER a success in rural Guatemala as evidenced by the increase in knowledge of the participants, increased prenatal visits performed in the region, and increased referrals of high-risk women. Of most significance is the increased recognition and referral of high-risk pregnancies as these are factors that inarguably improve maternal health outcomes (3). When TBA training programs fail in LMICs, it is due to lack of integration into the current health-care infrastructure and unwillingness of national health workers to accept the potential role of TBAs (32). Fortunately, these are factors that have not been heavily faced as the MSPAS has supported and helped to integrate the School of POWHER into the current health-care infrastructure from its conception.

In the current medical literature on birth attendant training programs, whether traditional or skilled, the reports on training programs is increasing but there are little data on how those programs directly affect clinical skills and access to prenatal care (4). Some studies have evaluated antenatal care skills yet they have focused on simulated sessions (33) and cross-sectional views on current prenatal skill set (34). Such studies do not provide a real-time look at how a training program directly affects the antenatal clinical skills of their trainees. In order to provide pertinent information lacking in the current literature and further integrate these indigenous health providers within the Guatemalan health-care system a real time, systematic assessment of the School of POWHER participants has been added to the clinical curriculum.

With the incorporation and implementation of a clinical skills checklist, there will be a sustainable quantitative measure of the capability in which participants of the School of POWHER are performing basic prenatal care during their routine home visits. Future applications for the skills checklist include an assessment of how many threshold home visits and/or total months of clinical education are required from each participant to optimize prenatal care. Additionally, the checklist will help to capture larger outcomes measures including number of referrals of expectant women to MSPAS health clinics, maternal and neonatal mortality, and other complications during pregnancy. Ultimately, by demonstrating to the MSPAS that participants can provide adequate prenatal care, refer appropriately, and adhere to their national guidelines, the School of POWHER is helping to create a standard of care for the comadrona practitioner.

The School of POWHER, of a school for comadronas run by comadronas, is critical. Integrating comadronas as leaders and educators will help shift the view some still hold of comadronas as a burden to the system (12) to actual players within the health-care infrastructure (13). Ultimately, the School of POWHER will further reiterate the pivotal role comadronas, and all TBAs in LMICs, hold as educators, knowledgeable birth attendants, and unique health-care providers.

## Lessons Learned/Recommendations

The need for sustainable TBA training programs in LMICs is much needed. TBAs have held, and will continue to hold, a very important role in their communities and their adequate training can help create the link between women in rural communities and the national health-care infrastructure. Yet, establishing this crucial link is a gradual process that faces some barriers and limitations.

Despite the long-standing collaboration between Saving Mothers and the MSPAS, participants in our School of POWHER continue to report some ongoing conflict with particular MSPAS health staff. Comadronas have described, during informal discussion sessions, that they are reprimanded and blamed for patient complications when they refer a high-risk patient to the local MSPAS health post or hospital. Although these interactions are isolated events they have the potential to affect comadrona attitudes toward certain physicians. These changes in attitudes might influence their decision on whether or not to refer their patients. With the recent implementation of the new primary health-care model in Guatemala, which acknowledges the role of the comadrona as a type of health-care provider, the negative attitudes toward comadronas, and instances of friction, might be decreasing. This case study is limited in that the viewpoint and/or opinions of the MSPAS on-call physicians are not presented. In future research, it would be of value to discuss, through focus groups and/or anonymous surveys, why certain physicians prefer that comadronas not be trained in basic prenatal care. These opinions would present a more complete picture of the actual health-care attitudes in rural Guatemala.

A limitation of this community case study is the quantity of reportable data available from the School of POWHER that can assist in measuring its objective success. From the beginning of the training program, knowledge acquirement and retention data were easily collected through written exams. Yet during the initial intervention, mainly due to lack of on the ground staff, amount of prenatal visits completed and number of high-risk patients referred from program participants was not wholly captured. As the educational intervention began to acquire larger amounts of funding, additional on the ground staff could be hired thereby increasing the amount of reportable data collected in 2016. Finally, with the launch of the 100% sustainable School of POWHER in early 2017, data collection by the eight comadrona educators will be an easier process during monthly follow-up education. This increase in data collection, specifically recorded prenatal visits as well as number and reasons of referrals by each participant, will help both Saving Mothers and the MSPAS gage the educational intervention’s direct effect on the health-care outcomes of women in the region.

Lessons learned on how to implement and sustain a training program for TBAs in rural Guatemala can be applied to other LMICs, specifically in indigenous populations. Implementation of the School of POWHER needs the dual support of the community as well as the Ministry of Health in each country using this model. Follow-up education, both for skills maintenance and data collection, is key and must be incorporated from the beginning. This community case study is one of the first to report on an educational model that is run by TBAs for TBAs in an indigenous rural population and demonstrates the importance of respecting cultural customs and traditions while building strong collaborative relationships between international and national partners.

## Conclusion

This community case study of the School of POWHER has documented the health deficit encountered in many LMICs, has discussed its methodological framework, proposed ways of evaluating its success, and ultimately demonstrated the importance and value added in training TBAs and having them train their counterparts. This immersive intervention has created a new model of TBA recruitment and training that instills a value of knowledge sharing and apprenticeship while emphasizing a strong partnership with local MSPAS providers. In essence, the School of POWHER is positioning comadronas as cultural medical brokers between indigenous women and the health-care system (35) ultimately increasing access to health care for women and reducing complications during pregnancy and labor with the potential to do the same in other LMICs.

## Author Contributions

SH assisted in the transition of the new model of the training program, acquired study data, and drafted, and revised the manuscript. JO contributed to the conception, initial development, and implementation of the training program. TS contributed to the initial conception and development of the training program and revised the manuscript. All authors approve the final manuscript.

## Conflict of Interest Statement

The authors declare that the research was conducted in the absence of any commercial or financial relationships that could be construed as a potential conflict of interest.
